# Microbial Translocation and Inflammation Occur in Hyperacute Immunodeficiency Virus Infection and Compromise Host Control of Virus Replication

**DOI:** 10.1371/journal.ppat.1006048

**Published:** 2016-12-07

**Authors:** Adam J. Ericsen, Michael Lauck, Mariel S. Mohns, Sarah R. DiNapoli, James P. Mutschler, Justin M. Greene, Jason T. Weinfurter, Gabrielle Lehrer-Brey, Trent M. Prall, Samantha M. Gieger, Connor R. Buechler, Kristin A. Crosno, Eric J. Peterson, Matthew R. Reynolds, Roger W. Wiseman, Benjamin J. Burwitz, Jacob D. Estes, Jonah B. Sacha, Thomas C. Friedrich, Jason M. Brenchley, David H. O’Connor

**Affiliations:** 1 Department of Pathology and Laboratory Medicine, University of Wisconsin-Madison, Madison, Wisconsin, United States Of America; 2 Virology Training Program, University of Wisconsin-Madison, Madison, Wisconsin, United States Of America; 3 Laboratory of Parasitic Diseases, National Institute of Allergy and Infectious Disease, National Institutes of Health, Bethesda, Maryland, United States Of America; 4 Wisconsin National Primate Research Center, Madison, Wisconsin, United States Of America; 5 Vaccine & Gene Therapy Institute, Oregon National Primate Research Center, and Department of Molecular Microbiology & Immunology, Oregon Health & Science University, Portland, Oregon, United States Of America; 6 AIDS and Cancer Virus Program, Frederick National Laboratory for Cancer Research, Leidos Biomedical Research, Inc., Frederick, Maryland, United States Of America; 7 Department of Pathobiological Sciences, University of Wisconsin-Madison, Madison, Wisconsin, United States Of America; Vaccine Research Center, UNITED STATES

## Abstract

Within the first three weeks of human immunodeficiency virus (HIV) infection, virus replication peaks in peripheral blood. Despite the critical, causal role of virus replication in determining transmissibility and kinetics of progression to acquired immune deficiency syndrome (AIDS), there is limited understanding of the conditions required to transform the small localized transmitted founder virus population into a large and heterogeneous systemic infection. Here we show that during the hyperacute “pre-peak” phase of simian immunodeficiency virus (SIV) infection in macaques, high levels of microbial DNA transiently translocate into peripheral blood. This, heretofore unappreciated, hyperacute-phase microbial translocation was accompanied by sustained reduction of lipopolysaccharide (LPS)-specific antibody titer, intestinal permeability, increased abundance of CD4+CCR5+ T cell targets of virus replication, and T cell activation. To test whether increasing gastrointestinal permeability to cause microbial translocation would amplify viremia, we treated two SIV-infected macaque ‘elite controllers’ with a short-course of dextran sulfate sodium (DSS)–stimulating a transient increase in microbial translocation and a prolonged recrudescent viremia. Altogether, our data implicates translocating microbes as amplifiers of immunodeficiency virus replication that effectively undermine the host’s capacity to contain infection.

## Author Summary

We infected macaques with simian immunodeficiency virus (SIV) to identify phenomena that may compromise immunological containment of HIV replication following transmission. Within the first days of infection, we detected a significant increase in plasma levels of microbial products. This ‘microbial translocation’ was accompanied by inflammation and expansion of the immune cells that the virus exploits to establish infection. We postulate that this hyperacute microbial event serves to promote virus replication during a period in which it would be vulnerable to the host immune response.

## Introduction

In HIV infection, there is mounting evidence that host-virus interactions occurring prior to peak viremia serve as key determinants of durable host containment of virus replication[1]. Despite the importance of these hyperacute phase phenomena in dictating the pace of virus dissemination and disease progression, there is limited understanding of actionable targets for manipulating the host environment in which these early interactions take place.

One of the hallmark features of chronic HIV and SIV infection is a persistent and pathogenic translocation of gastrointestinal microbial products into peripheral blood[2,3]. Although the consequences of microbial translocation are complex and multifaceted, when inoculated into healthy individuals, microbial products stimulate a marked expansion of the CD4+CCR5+ T cell compartment[4], which is the primary cell type in which HIV/SIV replicates and serves as a key determinant of early HIV viremia[5]. In cultured lamina propria mononuclear cells infected with HIV, exposure to microbial products stimulates virus replication[6]. Inoculation of bacterial LPS into non-human primates chronically infected with SIV provokes a striking logarithmic increase in plasma viremia[7,8]. Critically, microbial translocation does not appear to occur in ‘natural host’ species, such as Sooty Mangabeys and African green monkeys, in which SIV infection is non-pathogenic[9,10].

Given the demonstrated capacity of microbial products to promote immunodeficiency virus replication *in vitro* and *in vivo*, we hypothesized that translocation of microbial products into blood occurs early during pathogenic immunodeficiency infection and likely amplifies viremia.

## Results

### SIV infection of 8 MHC-identical macaques

**Fig 1:**
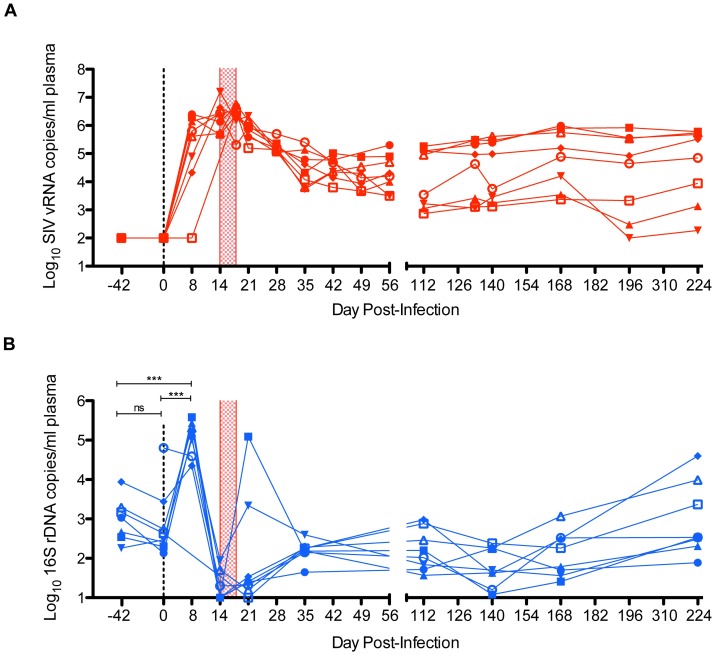
Longitudinal levels of SIV RNA and bacterial rDNA in plasma. Eight MHC-identical cynomolgus macaques became infected following intrarectal inoculation with SIVmac239. (**A**) The number of SIV RNA copies/ml of plasma was enumerated using qRT-PCR. Values are Log_10_-transformed and plotted longitudinally. (**B**) 16S sequencing data was used to correct raw 16S rDNA qPCR data by removing the proportion of 16S rDNA copies that corresponded to taxa detected in matched water controls. Corrected 16S rDNA copy data was Log_10_-transformed and plotted longitudinally. By Bonferroni-corrected one-way ANOVA, plasma levels of 16S rDNA did not change significantly between -42 and 0 DPI. Plasma levels of 16S rDNA increased significantly (P<0.0005) from both -42 to 8 DPI and 0 to 8 DPI. In both plots, the vertical checkered box positioned between 14 and 18 DPI corresponds to the acute-phase peak of SIV replication as detected by our sampling resolution.

We infected eight major histocompatibility complex (MHC)-identical cynomolgus macaques with molecularly cloned SIVmac239. Virus replication peaked at 14 (n = 3) or 18 (n = 5) days post-infection (DPI), reaching titers ranging from 6.4Log_10_ to 7.2Log_10_ viral RNA (vRNA) copies per milliliter (copies/ml) of plasma (Fig 1A). Acute-phase virus replication decreased to establish chronic-phase set-point viral loads, which were calculated as the geometric mean of 70–140 DPI viral loads, ranging from 3.2Log_10_ to 5.2Log_10_ vRNA copies/ml of plasma.

### Increased plasma titer of 16S ribosomal DNA

To monitor the quantity of microbial genomic DNA circulating in peripheral blood, we combined universal 16S ribosomal DNA (rDNA) sequencing with 16S rDNA qPCR[11]. To remove potentially confounding 16S rDNA contamination, which is commonly introduced by molecular biology reagents[12], we passed genera-level taxonomies through a correction workflow (S1 Fig) to bioinformatically remove genera from experimental samples if they were detected in contemporaneously prepared water controls.

Forty-two days prior to SIV infection, 16S rDNA loads averaged 2.9±0.5Log_10_ copies/ml of plasma (n = 7) **(** Fig 1B **)**. On the day of SIV challenge (0 DPI), 16S rDNA loads were not significantly different, averaging 2.8±0.8Log_10_ copies/ml (n = 8). At 8 DPI, in all but 1 animal, 16S rDNA loads increased between 4 and 1390 fold (5.0±0.4Log_10_ copies/ml; n = 7) above baseline. On average, 16S rDNA loads at 8 DPI were 421-fold higher than pre-infection levels. By 14 DPI, 16S rDNA loads decreased to near or below baseline levels, averaging 1.0±0.6Log_10_ copies/ml (n = 8). 16S rDNA loads remained stable between 21–168 DPI (2.0±1.4Log_10_ to 2.1±0.5Log_10_ copies/ml; n = 8). From 168–224 DPI, average plasma 16S rDNA loads increased (P = 0.0078; n = 8) to 2.9±0.9Log_10_ copies/ml, which are similar to levels observed during untreated HIV infection[13].

To determine whether this hyperacute-phase increase in microbial DNA was an artifact of our cynomolgus macaque model, we obtained 0 and 7 DPI plasma samples corresponding to an independent cohort (n = 11) of Indian rhesus macaques. These animals were infected following a single intravenous inoculation of swarm SIVmac251 inoculum. As in the cynomolgus macaque cohort, we found that 16S rDNA loads increased (P = 0.002; n = 11) from 0–7 DPI from 1.9±1.5Log_10_ to 3.4±1.1Log_10_ 16S rDNA copies/ml of plasma (S2A and S2B Fig **)**. In a cohort of mock-challenged macaques, we did not observe a significant (P = 1.000; n = 5) change in 16S rDNA loads between 0 and 7 days post-inoculation of phosphate-buffered saline (S2C Fig).

Taken together, these data identify a transient high magnitude increase in circulating levels of bacterial rDNA prior to peak SIV viremia, which is (a) not specific to a single species of macaque, (b) not unique to a single SIV challenge virus, and (c) not dependent on the route of transmission.

### Taxonomic characterization of microbial DNA in plasma

**Fig 2:**
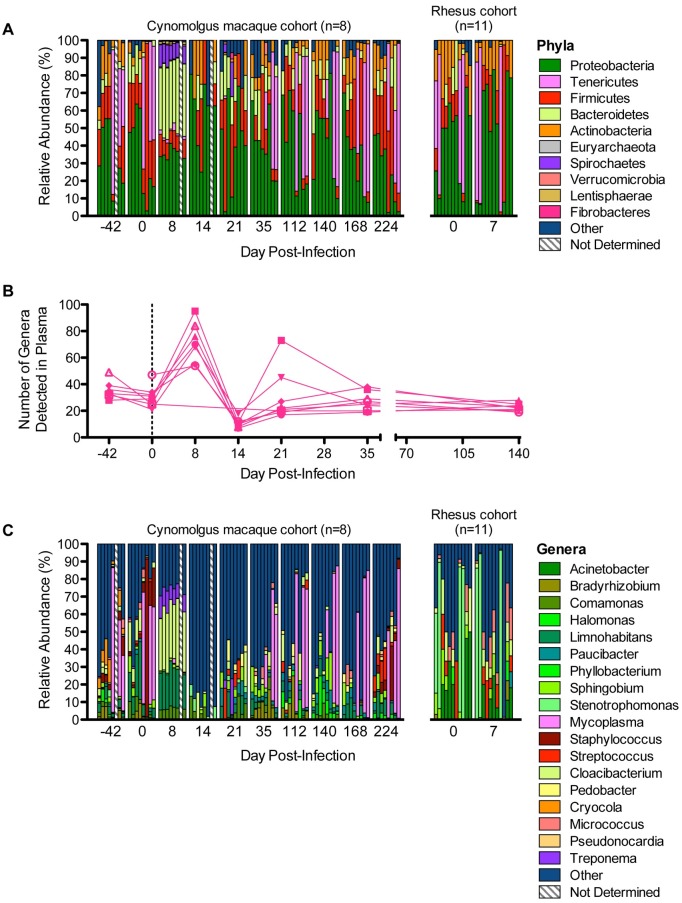
Taxonomic characterization of translocating microbial products. (**A**) Longitudinal relative abundance (%) of major phyla detected in blood plasma. (**B**) Number of unique bacterial genera for which genomic DNA was detected in blood plasma throughout the period of observation. Each line corresponds to a single animal. (**C**) Longitudinal relative abundance (%) of major genera detected in blood plasma. For (A and C), vertical bars within a given cluster (time-point) correspond to each individual animal, and colored segments correspond to the proportion of specific taxa. Owing to sample limitations, relative abundance of microbial taxa could not be determined for all animals at all time-points.

Following quantification of 16S rDNA in plasma, we sought to identify the taxa detected in plasma. At the phylum-level, peripheral blood was predominated by *Proteobacteria* (39.1%±21.3%), *Firmicutes* (18.7%±16.7%), *Tenericutes* (16.5%±28.5%), and *Bacteroidetes* (11.5%±12.3%) (Fig 2A). Prior to SIV challenge, *Proteobacteria* accounted for 34.7%±20.9% and 42.5%±22.8% of plasma microbial products in cynomolgus macaques and rhesus macaques, respectively. At 8 DPI, *Proteobacteria* continued to predominate (46.7%±34.5%) the plasma microbial composition of rhesus macaques, whereas *Proteobacteria* were displaced by *Bacteroidetes* (39.6±3.1% vs. 35.6±3.3%) in the cynomolgus macaque cohort.

At the genera level, an average of 32.9±7.6 unique taxa were detected in cynomolgus macaque plasma prior to SIV infection (Fig 2B). By 8 DPI, when 16S rDNA in plasma was highest (per our sampling resolution), the number of genera in plasma increased to an average of 71.7±14.8. By 14 DPI, diversity dropped precipitously to a cohort average of 10.9±3.6 genera. By 21 DPI, genera count increased to near pre-infection levels (cohort average = 30.5±19.3 genera) and demonstrated relative stability at 35 DPI (cohort average = 27.3±6.9 genera) and 140 DPI (cohort average = 22.8±2.9 genera).

Among the most abundant genera observed at 8 DPI were *Limnohabitans*, *Cloacibacterium*, *Ruminococcus*, *Oscillospira*, and *Lactobacillus* (Fig 2C). Although *Ruminococcus*, *Oscillospira*, and *Lactobacillus* are known constituents of cynomolgus macaque flora[14], *Cloacibacterium* and *Limnohabitans* are not often observed in vertebrates. However, *Cloacibacterium* has been observed in the gastrointestinal tract of patients with adenomas[15], and *Limnohabitans* has been observed in patients with cellulitis[16]**.** Both genera were detected at multiple timepoints, in multiple, but not all, animals. In rhesus macaques, the most abundant genera at 7 DPI were *Acinetobacter*, *Micrococcus*, *Phyllobacterium*, *Pseudonocardia*, and *Stenotrophomonas*. Future studies will be needed to better understand differences between cynomolgus macaques and rhesus macaques, specifically in regard to the composition of gastrointestinal microbial community and identity of translocating taxa.

### Stool microbial community and intestinal permeability

**Fig 3:**
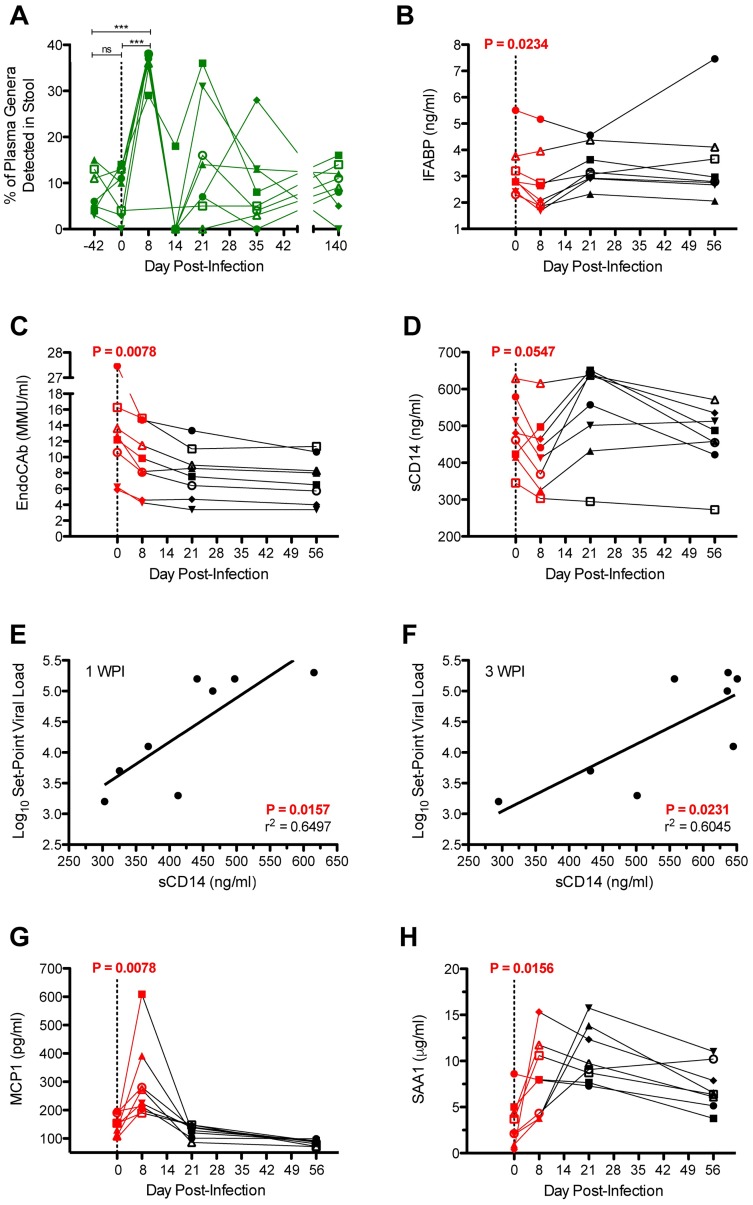
Plasma markers of intestinal breach and host response to microbial translocation. (**A**) Longitudinal proportion (%) of plasma genera detected in contemporaneous stool. Each line corresponds to a single animal. Bonferroni-corrected one-way ANOVA was used to calculate statistical significance. (**B**) Longitudinal plasma levels of IFABP, a marker of enterocyte loss and generalized damage to the intestinal epithelium. The host response to microbial translocation was measured using plasma levels of (**C**) EndoCAb, and (**D**) sCD14. By linear regression analysis, plasma levels of sCD14 at (**E**) 8 DPI, and (**F**) 21 DPI correlated positively with chronic-phase set-point viral loads. Acute inflammation was measured using plasma levels of (**G**) MCP-1, and (**H**) SAA1. For B, C, D, G, and H, differences between 0–8 DPI were evaluated for statistical significance by two-tailed Wilcoxon signed rank testing.

Following SIV challenge, we did not detect overt microbial dysbiosis within the stool of cynomolgus macaques. Across all timepoints, four phyla were predominant in stool–*Proteobacteria*, *Firmicutes*, *Bacteroidetes*, and *Spirochaetes* (S3A Fig). At the genera-level, *Prevotella* and *Treponema* were maintained at high abundance throughout the period of observation (S3B Fig). Although this study was not designed to identify the origin of translocating microbial products, the gastrointestinal tract has been reported as a major source of translocation in HIV/SIV infection[2]. To explore the relationship between microbial translocation and the gastrointestinal microbial community, we calculated the proportion of circulating bacterial rDNA corresponding to genera detected in contemporaneous stool (Fig 3A). We found that following SIV challenge, the overlap between microbial DNA sequences in plasma and stool increased significantly (P<0.0005; n = 7), which suggests that at least some of the microbial products translocating into peripheral blood originated from the gastrointestinal tract. However, following SIV challenge, we observed a decrease in plasma levels of intestinal fatty acid binding protein (IFABP), which is released into systemic circulation[17] by enterocytes when intestinal epithelium is compromised. From 0–8 DPI, plasma IFABP decreased (P = 0.0234; n = 8) from 3.2±1.0 ng/ml to 2.7±1.2 ng/ml (Fig 3B). By 21 DPI, IFABP levels (3.4±0.8 ng/ml; n = 8) were not significantly different from baseline. These data suggest that some proportion of hyperacute microbial translocation may originate from the gastrointestinal tract, but that there is not significant loss of intestinal epithelium integrity.

### Translocation-specific host response

We next quantified plasma levels of bacteria-specific host factors within the cohort of 8 MHC-identical cynomolgus macaques infected with SIVmac239. In the context of microbial translocation, host antibodies specific for bacterial endocore (EndoCAb) bind and clear LPS from circulation, which consequently reduces their titer[18]. From 0–8 DPI, cohort average EndoCAb levels decreased significantly (P = 0.0078; n = 8) from 13.1±6.7 Milli Merck Units (MMU)/ml to 9.5±4.0 MMU/ml (Fig 3C). This reduction is consistent with saturation of circulating EndoCAb by translocating LPS.

Although not strictly a measure of the bacteria-specific host response[19], sCD14 circulates at high levels in the plasma of healthy individuals[20] and interacts with translocating LPS to stimulate antigen-presenting cells via toll-like receptor signaling[21]. We did not observe a statistically significant change (P = 0.0547; n = 8) in circulating levels of sCD14 from 0 DPI (480.9±91.7 ng/ml) to 8 DPI (428.6±100.9 ng/ml) (Fig 3D), which is consistent with previous observations[22]. During chronic HIV infection, plasma levels of sCD14 are known to predict the relative rate of progression to AIDS[23]. Consistent with this prognostic association, we found that plasma levels of sCD14 at both 8 DPI (r^2^ = 0.6497, P = 0.0157; n = 8) and at 21 DPI (r^2^ = 0.6045, P = 0.0231; n = 8) correlated with set-point viral load during chronic infection (Fig 3E & 3F). We found no relationship between plasma levels of sCD14 and EndoCAb (S4 Fig).

### Hyperacute immune activation

We next assessed the relationship between hyperacute microbial translocation and peripheral inflammation. One of the earliest host responses to HIV infection is an increase in plasma levels of monocyte chemotactic protein 1 (MCP-1)[24], which is secreted by various cell types to recruit lymphocytes and phagocytes to sites of inflammation[25]. From 0–8 DPI, we observed a significant increase (P = 0.0078; n = 8) in MCP-1 levels from 147.1±34.4 pg/ml to 297.7±141.4 pg/ml (Fig 3G). By 21 DPI, MCP-1 levels had decreased to below baseline (126.2±22.6 pg/ml; n = 8), and continued to decrease at 56 DPI (85.5±9.9 pg/ml; n = 8). Serum amyloid A (SAA1), which is produced in the liver, is commonly used to measure relative levels of inflammation[26] and promotes chemotaxis of both lymphocytes and phagocytes[27]. From 0–8 DPI, plasma levels of SAA1 increased (P = 0.0156; n = 8) from 3.4±2.7 μg/ml to 8.2±4.2 μg/ml (Fig 3H). The abundance of CD4+CCR5+ T cells, which are the primary targets of HIV/SIV replication[28], increases during acute SIV infection in Asian-origin macaques[29] as well as following exposure to bacterial LPS[4]. We used flow cytometry (S5A Fig) and complete blood counts (CBCs) to monitor CD4+CCR5+ target cell abundance in peripheral blood. From 0–8 DPI, we found that the frequency of CD4+ cells expressing CCR5 increased (P = 0.0078; n = 8) from 1.5±0.3% to 3.6±1.0% (S5B Fig). By 21 DPI, the average peripheral frequency of CD4+ cells expressing CCR5+ increased to 4.8±2.4%. From 0–8 DPI, the absolute number of CD4+CCR5+ cells increased (P = 0.0313; n = 8) transiently from 10.1±3.2 to 26.0±11.7 cells per μl blood (S5C Fig). By 21 DPI, the number of CD4+CCR5+ cells decreased to 18.6±7.0 cells. We interpret these dynamics as hyperacute expansion of the CD4+CCR5+ target cell compartment with subsequent contraction into the chronic phase of infection due to a combination of virus replication and withdrawal of microbial products. Longitudinal CD4 counts and peripheral levels of Ki67+, CD38+, and HLA-DR+ subsets of CD4+ and CD8+ T cells are shown in S5D–S5J Fig.

### Increased SIV viremia following chemical induction of gastrointestinal distress and microbial translocation

**Fig 4:**
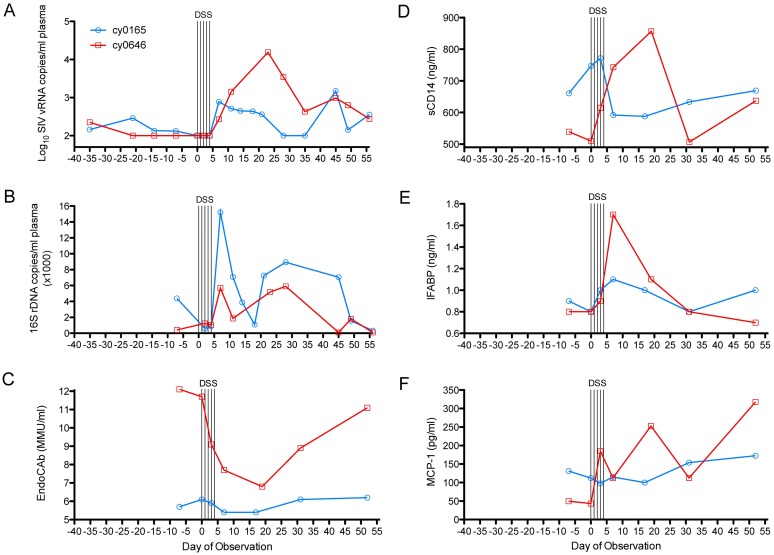
Chemically inducing microbial translocation stimulates multiple host inflammatory processes and increases plasma viremia and levels of bacterial rDNA. (**A**) Log_10_-transformed plasma SIV load. (**B**) Linear plasma 16S rDNA load. The bacteria-specific host response was assessed by monitoring plasma levels of (**C**) EndoCAb and (**D**) sCD14. Plasma IFABP levels (**E**) were used to monitor changes to the integrity of the gastrointestinal epithelium. Generalized inflammation was monitored using plama levels of (**F**) MCP-1. In all panels, 5 black vertical lines indicate the 5-day period of once-daily treatment with dextran sulfate sodium (DSS).

We selected two “elite controller” cynomolgus macaques that had maintained average plasma viral loads of 2.2±1.9Log_10_ (for cy0165) and 2.1±1.7Log_10_ (for cy0646) vRNA copies/ml of plasma across a 5-week period of baseline observation (Fig 4A). To increase gastrointestinal permeability and facilitate microbial translocation, we treated both macaques with dextran sulfate sodium (DSS) as described previously[30]. Briefly, both macaques were gavaged with 200 ml of sterile drinking water containing 0.5% DSS once per day for 5 consecutive days (on days 0, 1, 2, 3, and 4 of observation). During treatment, both animals maintained plasma viremia to levels below the limit of detection. By day 7 of observation (day 3 post-DSS treatment), plasma viremia increased in both animals to 2.4 Log_10_ and 2.9Log_10_ vRNA copies/ml plasma. In one of the animals, cy0165, plasma viremia decreased gradually until day 28 of observation. The effect of treatment was more significant in the second animal, cy0646. From day 7 to 11 of observation, plasma viremia increased in titer to 3.2Log_10_ vRNA copies/ml. Due to blood draw volume limits, combined with sudden weight loss (S6A Fig), we were unable to monitor plasma viremia in cy0646 from day 12 to 22 of observation. By day 23 of observation, plasma viremia for cy0646 had increased to 4.2Log_10_ vRNA copies/ml plasma– corresponding to more than a 120-fold increase over pre-treatment SIV titer. By day 28, plasma viremia had decreased to 3.5Log_10_ vRNA copies/ml, and continued to decrease until day 34 of observation. Despite variation in the dynamics by which virus recrudesced, both animals experienced a marked prolonged increase in viremia following one short-course DSS treatment cycle.

### Increased 16S rDNA load following chemical induction of gastrointestinal distress and microbial translocation

Prior to starting DSS treatment (day -7 of observation), the macaques had plasma 16S rDNA loads of 4,380 and 420 copies/ml of plasma for cy0165 and cy0646, respectively (Fig 4B). During DSS treatment (day 2 and 4 of observation), 16S rDNA loads averaged 740±198 copies/ml and 1130±141 copies/ml for cy0165 and cy0646, respectively. By day 7 of observation (day 3 following cessation of DSS treatment), 16S rDNA loads had increased to 15,220 copies/ml and 5,690 copies/ml for cy0165 and cy0646, respectively. Consistent with our hyperacute-phase observations, the observed increase in plasma 16S rDNA load was transient, having decreased in both animals to near pre-treatment titers by day 11 of observation. One of the animals, cy0646, experienced significant weight loss following DSS treatment and could not be sampled between day 12 and 22 of observation. Despite this undesirable clinical effect, the increased plasma 16S rDNA titer following DSS treatment was markedly lower than that observed in hyperacute SIV infection.

### Perturbation of translocation-specific host factors, peripheral inflammation, and gastrointestinal permeability following chemical induction of gastrointestinal distress and microbial translocation

We first examined plasma levels of bacteria-reactive host factors– EndoCAb and sCD14. Prior to DSS treatment, plasma EndoCAb levels (Fig 4C) were 5.7±0.43 MMU/ml and 12.1±0.7 MMU/ml for cy0165 and cy0646, respectively. Following treatment, cy0165’s EndoCAb levels did not change markedly, though decreased precipitously in cy0646 to 6.8 MMU/ml by day 19 of observation. The marked decrease in circulating EndoCAb for cy0646 is consistent with it being saturated by translocating LPS. Prior to treatment, plasma sCD14 levels (Fig 4D) were 661±77 **ng/ml** and 539±15 **ng/ml** for cy0165 and cy0646, respectively. Following treatment, sCD14 levels increased in cy0165 to 772 ng/ml (1.7-fold increase) by day 7 of observation. More delayed kinetics were observed for cy0646, though sCD14 levels increased to 857 ng/ml (1.6-fold over baseline) by day 19 post-treatment. Again, we used plasma levels of IFABP to mark increased gastrointestinal permeability (Fig 4E). Relative to baseline, post-treatment IFABP levels increased as much as 1.16-fold (to 1.1 ng/ml) and 2.22-fold (to 1.7 ng/ml) for cy0165 and cy0646, respectively. These results implicate DSS-mediated dysfunction of gut barrier integrity in our observed increase in plasma viremia and circulating 16S rDNA. We next measured plasma levels of inflammatory factor, MCP-1 (Fig 4F). Prior to treatment, cy0165 and cy0646 maintained plasma MCP-1 levels of 131.2±18.3 pg/ml and 49.8±58.5 pg/ml, respectively. MCP-1 levels did not change markedly for cy0165 at any timepoint, but cy0646 showed fluctuating levels until day 52, at which time MCP-1 levels had increased to 317.8 pg/ml (6.4-fold above baseline). Altogether, DSS treatment stimulated increased inflammation, increased intestinal permeability, and perturbed translocation-reactive host factors.

## Discussion

The systemic dissemination and replication of certain enteric viruses has been tied to their interaction with gastrointestinal bacteria[31]. In the gastrointestinal tract, mouse mammary tumor virus, an orally transmitted retrovirus, becomes coated in bacterial LPS, which causes circulating virions to provoke interleukin-10 production, which aids in virus subversion of cellular immunity[32]. Intriguingly, elicitation of anti-gp41 antibodies in a recent HIV vaccination study was purportedly compromised by epitope sharing between commensally encoded antigen and the viral gp41 glycoprotein[33]. These data, when considered alongside evidence that gastrointestinal barrier integrity is compromised early during HIV infection[34], point toward microbial products being beneficial to incipient HIV infection.

When bacterial LPS is inoculated into healthy human volunteers, it stimulates a marked increase in the availability of CD4+CCR5+ T cells[4]. Given that CCR5-tropic variants of HIV are responsible for newly transmitted HIV infections[35,36], it is tempting to speculate that bacterial products in circulation during early HIV infection promote virus replication, both directly by stimulating CD4+CCR5+ target cell compartment expansion, and indirectly by generally increasing immune activation. This hypothesis is supported by the observation that disease severity is attenuated in SIV-infected macaques that are treated with the LPS-binding drug, sevelamer, during acute infection[37], as well as the data presented in this manuscript showing that inducing microbial translocation stimulates increased viremia in SIV-infected macaques.

In our study, prior to the acute-phase peak of viremia, we observed a high-magnitude increase in circulating levels of 16S rDNA. We can only speculate that the observed increase in 16S rDNA titer corresponds to increased entry of either intact bacteria or dissociated bacterial products into circulation. Alternatively, the increase in 16S rDNA titer may correspond to impaired host clearance of pre-existing bacteria (or dissociated products) in blood or tissues. However, the rapid reduction of 16S rDNA titer by 14 DPI suggests that host clearance of bacterial products was not particularly compromised during hyperacute SIV infection. Since the observed increase in 16S rDNA titer appears transient, if it corresponds to intact “live” bacteria, they may have been incapable of persisting within blood or tissues. Additionally, with regard to the transience of the observed hyperacute microbial translocation, enteropathy is known to occur during acute immunodeficiency virus infection. We suspect that as peak viremia declines to establish the chronic-phase set-point, with partial control of virus replication comes partial healing of the enteric lesions that facilitated translocation of microbial products from the gastrointestinal lumen. This would explain why the influx of 16S rDNA into blood and corresponding perturbation of inflammatory markers are transient. Although future studies incorporating longitudinal examination of the integrity of the gastrointestinal mucosa will be needed to confirm this speculation, this explanation is consistent with the transience of microbial translocation observed following short-course DSS treatment. It is also worth noting that plasma 16S rDNA loads likely underestimate the presence of 16S rDNA within whole blood[38]. Regardless, we found that increased 16S rDNA loads were accompanied by (a) prolonged saturation of LPS-specific antibodies, (b) transient perturbation of sCD14, increased levels of inflammation as measured by MCP-1 and SAA1, and (c) expansion of the peripheral CD4+CCR5+ T cell compartment. We also identified a correlation between acute-phase levels of sCD14 in plasma and chronic-phase plasma viral loads, which links chronic-phase viremia to hyperacute-phase phenomena.

Future study is required to determine whether gut barrier dysfunction occurs as a direct consequence of viral particles interacting with gastrointestinal epithelia[39] or an indirect consequence of the inflammatory response to incipient infection. Though mechanistic details have not yet been elucidated, our data suggest that incipient SIV infection compromises the integrity of the gastrointestinal epithelium, which led to our hypothesis that microbial translocation amplifies early immunodeficiency virus replication. Our hypothesis is supported by previous work[7,8,30,37,40] linking microbial exposure to increased plasma viremia and by our observation that a prolonged recrudescent viremia is provoked by short-course treatment of SIV-infected macaques with the gastrointestinal permeabilizing compound, DSS.

Our use of DSS has limitations that merit discussion. Although short-course DSS treatment did well to recapitulate the transience of microbial translocation observed in hyperacute SIV infection, the magnitude by which microbial products translocated was considerably higher during hyperacute SIV infection. Administering DSS at a higher dose might have more closely modeled this magnitude, but doing so may have jeopardized the health of our animals. Although exposure to microbial products[7,8,30,37,40] and oral administration of DSS[30] is known to increase immunodeficiency virus replication, our mechanistic understanding of how either insult amplifies viremia is incomplete. It is also important to note that characterizing the effect of specific translocating taxa will be imperative to better understanding both the fundamental and clinical implications of microbial translocation during immunodeficiency virus infection– this understanding will very likely require inoculating animals with well-defined assemblages of microbes. Since there is variation in plasma 16S rDNA titer at baseline, better understanding how an animal’s typical microbial “state” influences immunodeficiency virus infection may prove useful.

Despite the benefits of using SIV-infected macaques to model HIV infection, differences exist between macaques and humans that may be relevant to the hyperacute-phase of infection[41]. Although evidence exists[42], it is not currently clear whether microbial translocation occurs in hyperacute HIV infection, but studying early HIV infection requires longitudinally sampling people likely to become infected, which is ethically complicated given the demonstrated efficacy of pre-exposure prophylaxis.

In summary, we report hyperacute-phase microbial translocation as one of the earliest pathological events to occur during immunodeficiency virus infection, and that it may, in fact, precede detectable lesions along the gastrointestinal epithelium. Although future study, incorporating longitudinal examination of the gastrointestinal epithelium during hyperacute SIV infection, is needed to better resolve the precise kinetics of mucosal damage, we tested the hypothesis that experimentally compromising the barrier integrity of the gastrointestinal epithelium and inducing microbial translocation in SIV-infected macaques would compromise host control of virus replication. This finding has important implications for understanding interactions between immunodeficiency virus replication and the remarkable host responses capable of durably constraining progressive infection. HIV prophylaxis may benefit from incorporating strategies to mitigate the capacity for microbial products to amplify virus replication.

Lastly, a number of strategies are being investigated to provoke production of viral antigen from latently infected cells. Our DSS treatment data demonstrates that increasing gastrointestinal permeability and inducing microbial translocation results in a period of prolonged viremia, which suggests that inducing controlled rounds of microbial translocation (or mimicking microbial antigenemia) may be an effective strategy to reactivate the latent reservoir.

## Methods

### Animals and viral infections

Nine cynomolgus macaques (8 females and 1 male) were infected with SIV following a single atraumatic intrarectal inoculation with 7,000 TCID_50_ of SIVmac239 virus (Genbank: M33262). Five SIV-negative female cynomolgus macaques were mock-challenged by a single atraumatic intrarectal inoculation with phosphate-buffered saline. Eleven Indian-origin rhesus macaques were infected with SIV following intravenous-administration of SIVmac251 (500 TCID_50_) swarm inoculum.

### Chemical induction of microbial translocation

A 0.5% solution of dextran sulfate sodium (DSS) was prepared by resuspending colitis-grade DSS (MPBio, Santa Ana, CA) in sterile drinking water and stored at 4°C. To increase permeability of the gastrointestinal epithelium, animals were treated once per day for 5-consecutive days with 200ml of the DSS-containing drinking water– administered by gavage. Animals were monitored for clinical signs of colitis and gastrointestinal distress and received palliative and clinical care at the full discretion of WNPRC veterinarians.

### Plasma isolation, nucleic acid extraction, and SIV viral load quantification

Plasma was isolated from whole blood by ficoll-based density centrifugation, and cryopreserved at -80°C. Nucleic acid for each animal at each time-point was isolated from 300 μl of thawed cryopreserved plasma using the Maxwell 16 Viral Total Nucleic Acid Purification Kit (Promega, Madison WI), per the manufacturer’s specifications. This extraction method indiscriminately isolates both DNA and RNA, which we eluted into 50 μl of nuclease-free water. Nucleic acid was isolated from all eight (for the hyperacute study) or two (for the DSS treatment study) cynomolgus macaques within the same instrument run and using the same batch of reagents. At each time-point, water controls were processed alongside experimental samples. Following isolation, plasma nucleic acid was aliquoted into the SIV viral load assay, and the remainder was stored at -80°C until 16S-based sequencing and qPCR. Plasma SIV viral loads were determined as published previously[43].

### 16S ribosomal DNA sequencing, contaminant correction, and quantification

16S ribosomal PCR and sequencing were performed as previously described[44,45]**.** Briefly, the V4 region of the 16S ribosome gene was amplified from plasma DNA using barcoded Illumina-specific primers. Each sample was amplified in triplicate, pooled, and separated by 1% gel electrophoresis. After gel purification, samples were quantified with a Qubit high-sensitivity DNA kit (Invitrogen) and equimolar amounts of each sample were combined into a final pool. To exclude short fragments, the combined final pool was cleaned using the Ampure XP kit (Agilent, Santa Clara, CA). The final pool was then quantified and sequenced on the Illumina MiSeq to a depth sufficient to capture the total taxonomic composition of our samples, as determined by rarefaction. We used MacQIIME 1.9.1[46] to process the raw sequencing data to genera-level resolution using the default 97% identity similarity threshold for assembling operational taxonomic units (OTUs).

For each experimental and control sample, genera-level relative abundance OTU tables were generated and exported. Contaminant correction was performed by comparing genera-level OTUs present in experimental samples to those present in their corresponding water control. All genera detected (>0%) within sample-matched water controls were removed (frequency set to 0%) from the sample’s OTU table. The frequencies of remaining genera were then normalized to account for the proportion of genera removed from the sample’s microbial community.

To quantify the number of 16S ribosome DNA copies per milliliter of plasma, the absolute copy number was determined using a previously published universal 16S rDNA qPCR assay[11], and then corrected by subtracting the proportion of 16S rDNA copies that corresponded to contaminants. Differences between pre-infection and 8 DPI levels of bacterial 16S rDNA were evaluated for statistical significance using two-tailed Wilcoxon signed rank testing.

### Plasma markers of microbial translocation, immune activation, and intestinal damage

Plasma SAA1 and IFABP were quantified using a commercially available Monkey SAA1 ELISA kit (MyBioSource, San Diego, CA) and Monkey IFABP/FABP2 ELISA kit (MyBioSource, San Diego, CA) with 1:1000 and 1:2 sample dilution, respectively. Commercially available ELISA kits for Human CCL2/MCP-1 (R&D Systems, Minneapolis, MN), Human EndoCab IgM (Hycult Biotech, Plymouth Meeting, PA), and Human sCD14 (R&D Systems, Minneapolis, MN) were used according to the manufacturer’s protocols on plasma diluted 1:3, 1:100 and 1:300, respectively. Each sample was quantified in duplicate and analyzed with a 4-Parameter Logistic fit using the SoftMax Pro 6.4 program (Molecular Devices, Sunnyvale, CA). Differences between 0 and 8 DPI levels were evaluated for statistical significance using two-tailed Wilcoxon signed rank testing.

### Immunophenotyping with flow cytometry

Cryopreserved PBMCs were thawed at 37°C and washed in R10 media (RPMI containing 10% FBS). Between 3–5 million washed cells were transferred to 1.2 ml cluster tubes, and resuspended in 200 μl of R10 media. Cells were stained with 5 μl anti-CCR5-BV421 (clone 2D7/CCR5; BD Biosciences, San Jose, CA) at 37°C for 15 min. Cells were then surface-stained with 5 μl anti-CD4-PE/Cy7 (clone L200; Fisher Scientific) for 30 min at room temperature. Cells were washed twice with FACS buffer (PBS containing 10% FBS), fixed with 2% paraformaldehyde (PFA), incubated at 4°C, and run on a SORP BD-LSRII (BD Biosciences, San Jose, CA). FlowJo (version 9.8.3, Tree Star) was used to perform sample compensation and gating. For each sample, the frequency of peripheral CD4+CCR5+ cells was determined by first gating on singlet events, followed by gating on lymphocyte-sized cells, gating on CD4+ cells, and finally by gating on CCR5+ cells within the CD4+ gate. Differences between 0 and 8 DPI levels were evaluated for statistical significance using two-tailed Wilcoxon signed rank testing.

### Ethics statement

The cynomolgus macaques used in this study were cared for by the staff at the Wisconsin National Primate Research Center according to regulations and guidelines of the University of Wisconsin Institutional Animal Care and Use Committee, which approved this study (protocol g00517) in accordance with recommendations of the Weatherall report and according to the principles described in the National Research Council’s Guide for the Care and Use of Laboratory Animals. The Indian-origin rhesus macaques used in this study were cared for by the staff at the Oregon National Primate Research Center according to regulations and guidelines of the Oregon Health Sciences University Institutional Animal Care and Use Committee (protocol #0989) in accordance with recommendations of the Weatherall report and according to the principles described in the National Research Council’s Guide for the Care and Use of Laboratory Animals. Per Animal Wellfare Approved regulations, all animals were housed in enclosures with at least 4.3, 6.0, or 8.0 sq. ft. of floor space, measuring 30, 32, or 36 inches high, and containing a tubular PVC or stainless steel perch. Each individual enclosure was equipped with a horizontal or vertical sliding door, an automatic water lixit, and a stainless steel feed hopper. All animals were fed using a nutritional plan based on recommendations published by the National Research Council. Twice daily the macaques on the described studies were fed a fixed formula, extruded dry diet (2050 Teklad Global 20% Protein Primate Diet) with adequate carbohydrate, energy, fat, fiber (10%), mineral, protein, and vitamin content. Feeding strategies were individually tailored to the age and physical condition of the experimental subjects. Dry diets were supplemented with fruits, vegetables, and other edible objects (e.g., nuts, cereals, seed mixtures, yogurt, peanut butter, popcorn, marshmallows, etc.) to provide variety to the diet and to inspire species-specific behaviors such as foraging. To further promote psychological well-being, animals were provided with food enrichment, human-to-monkey interaction, structural enrichment, and manipulanda. Environmental enrichment objects were selected to minimize chances of pathogen transmission from one animal to another and from animals to care staff. While on study, all animals were evaluated by trained animal care staff at least twice each day for signs of pain, distress, and illness by observing appetite, stool quality, activity level, physical condition. Animals exhibiting abnormal presentation for any of these clinical parameters were provided appropriate care by attending veterinarians. Prior to all experimental procedures, animals were sedated using ketamine anesthesia, which was reversed at the conclusion of a procedure using atipamizole. Animals were monitored regularly until fully recovered from anesthesia. Animals were not euthanized as part of these studies.

## Supporting Information

S1 Fig Workflow for correcting plasma samples for reagent and environmental microbial contamination. (PDF)

S2 Fig Microbial translocation in SIV-infected rhesus macaques and mock-challenged cynomolgus macaques. (**A**) The number of SIV RNA copies/ml of plasma was enumerated using qRT-PCR. Values are Log_10_-transformed. (**B**) Plasma levels of 16S rDNA in SIVmac251-infected Indian rhesus macaques were enumerated by contaminant-correcting raw 16S rDNA qPCR data by removing the proportion of 16S rDNA copies that corresponded to genera detected in water controls. Corrected numbers of 16S rDNA copies are Log_10_-transformed. (**C**) Plasma levels of 16S rDNA in cynomolgus macaques mock-challenged by intrarectal inoculation with PBS.
(PDF)

S3 Fig Taxonomic characterization of the stool microbial community. (**A**) Longitudinal phylum-level identity of microbial genomic DNA in stool. (**B**) Longitudinal number of unique bacterial genera for which genomic DNA was detected in stool. For (A and B), vertical bars within a given cluster (time-point) correspond to each individual animal, and colored segments correspond to the proportion of specific taxa. Owing to sample limitations, relative abundance of microbial taxa could not be determined for all animals at all time-points.
(PDF)

S4 Fig No correlation between plasma levels of soluble CD14 and EndoCAb. (PDF)

S5 Fig The T cell compartment during SIV infection. (**A**) Representative FACS plots gated on peripheral CD4+ cells expressing the HIV/SIV co-receptor CCR5. (**B**) Frequency of peripheral CD4+ cells expressing CCR5. These frequencies were combined with complete blood counts to determine the (**C**) absolute number of CD4+CCR5+ cells per μl of blood. (**D**) Frequency of CD4+ cells expressing Ki67. (**E**) Frequency of CD8+ cells expressing Ki67. (**F**) Frequency of CD4+ cells expressing CD38. (**G**) Frequency of CD8+ cells expressing CD38. (**H**) Frequency of CD4+ cells expressing HLA-DR. (**I**) Frequency of CD8+ cells expressing HLA-DR. (**J**) CD4+ T cell count per microliter of peripheral blood. For all measures of statistical significance, differences between levels from 0 to 8 DPI were evaluated for statistical significance by two-tailed Wilcoxon signed rank testing.
(PDF)

S6 Fig Weight and hematological health during dextran sulfate sodium treatment. We monitored the weight (**A**) and hematological health (**B and C**) of both macaques during dextran sulfate sodium treatment.
(PDF)
