# Supplementary figures and images for: Microbial Translocation and Inflammation Occur in Hyperacute Immunodeficiency Virus Infection and Compromise Host Control of Virus Replication
Source: PLoS Pathog. 2016 Dec 7;12(12):e1006048. doi: 10.1371/journal.ppat.1006048 (PMC5142784; doi:10.1371/journal.ppat.1006048)

# SUP FIGURE 1

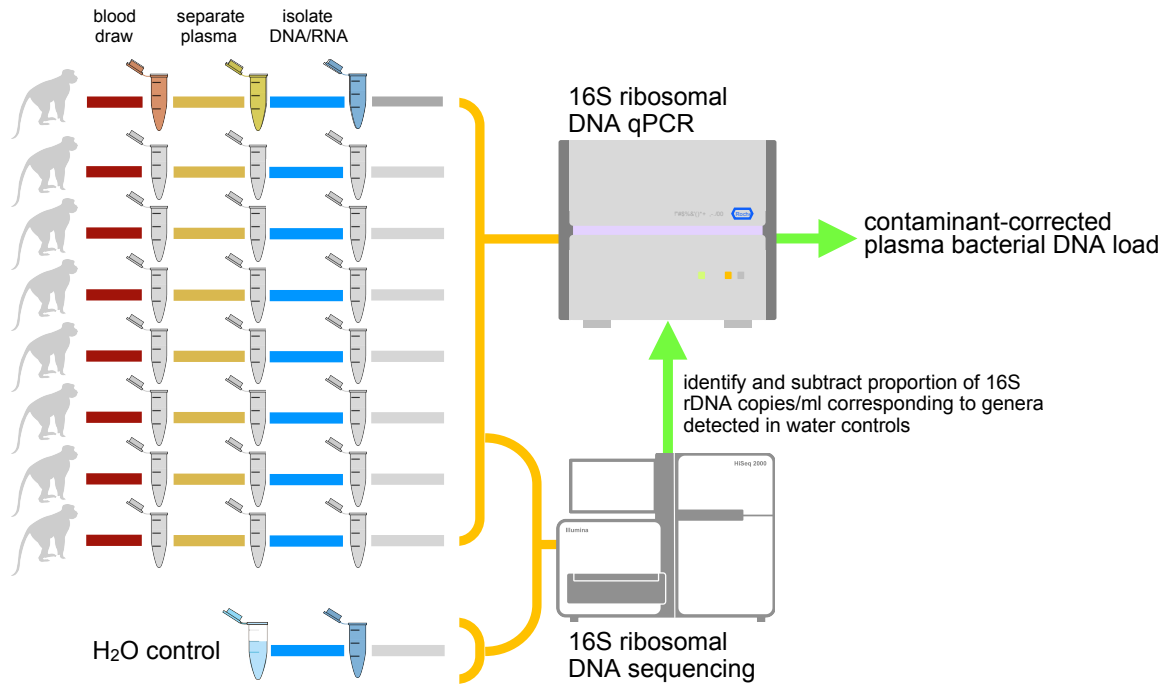

Supplement: S1 Fig — (PDF) [file ppat.1006048.s001.pdf]

## SUP FIGURE 2

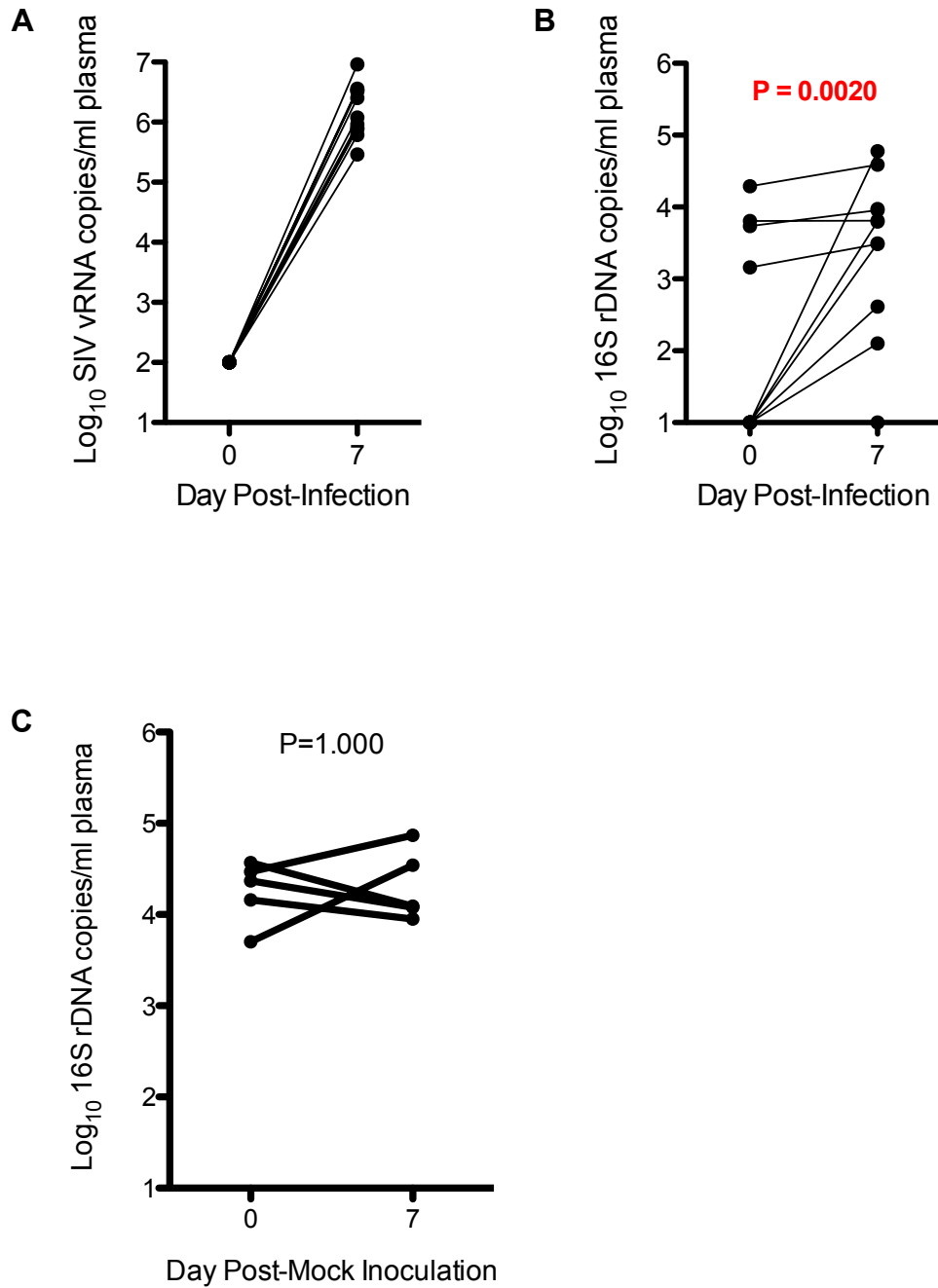

Supplement: S2 Fig — (A) The number of SIV RNA copies/ml of plasma was enumerated using qRT-PCR. Values are Log10-transformed. (B) Plasma levels of 16S rDNA in SIVmac251-infected Indian rhesus macaques were enumerated by contaminant-correcting raw 16S rDNA qPCR data by removing the proportion of 16S rDNA copies that corresponded to genera detected in water controls. Corrected numbers of 16S rDNA copies are Log10-transformed. (C) Plasma levels of 16S rDNA in cynomolgus macaques mock-challenged by intrarectal inoculation with PBS. (PDF) [file ppat.1006048.s002.pdf]

# SUP FIGURE 3

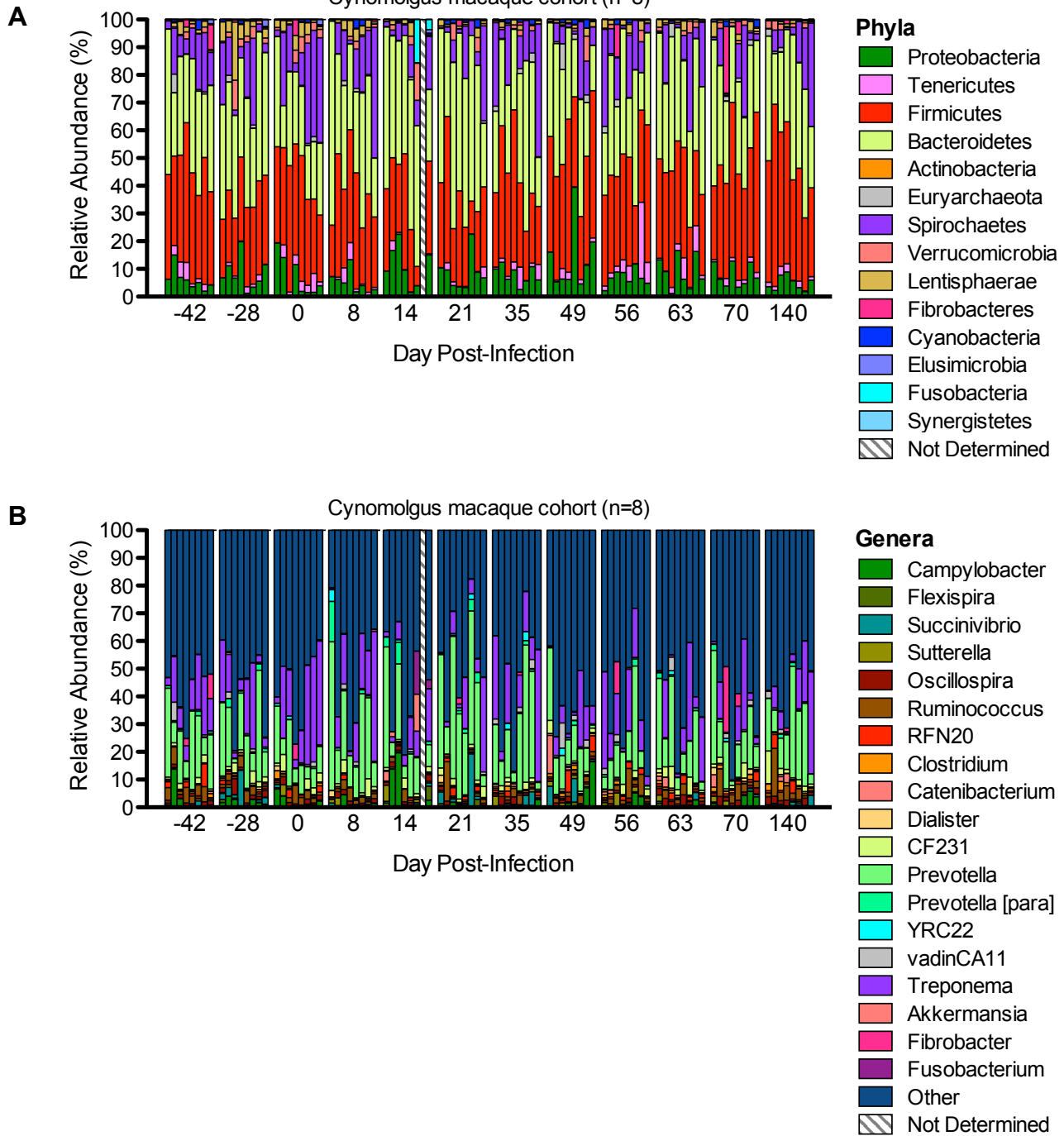

Supplement: S3 Fig — (A) Longitudinal phylum-level identity of microbial genomic DNA in stool. (B) Longitudinal number of unique bacterial genera for which genomic DNA was detected in stool. For (A and B), vertical bars within a given cluster (time-point) correspond to each individual animal, and colored segments correspond to the proportion of specific taxa. Owing to sample limitations, relative abundance of microbial taxa could not be determined for all animals at all time-points. (PDF) [file ppat.1006048.s003.pdf]

## SUP FIGURE 4

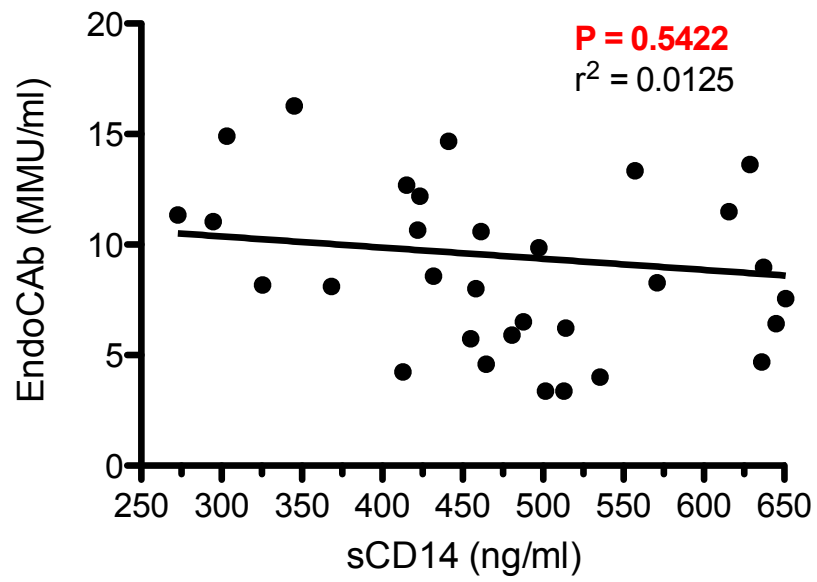

Supplement: S4 Fig — (PDF) [file ppat.1006048.s004.pdf]

# SUP FIGURE 5

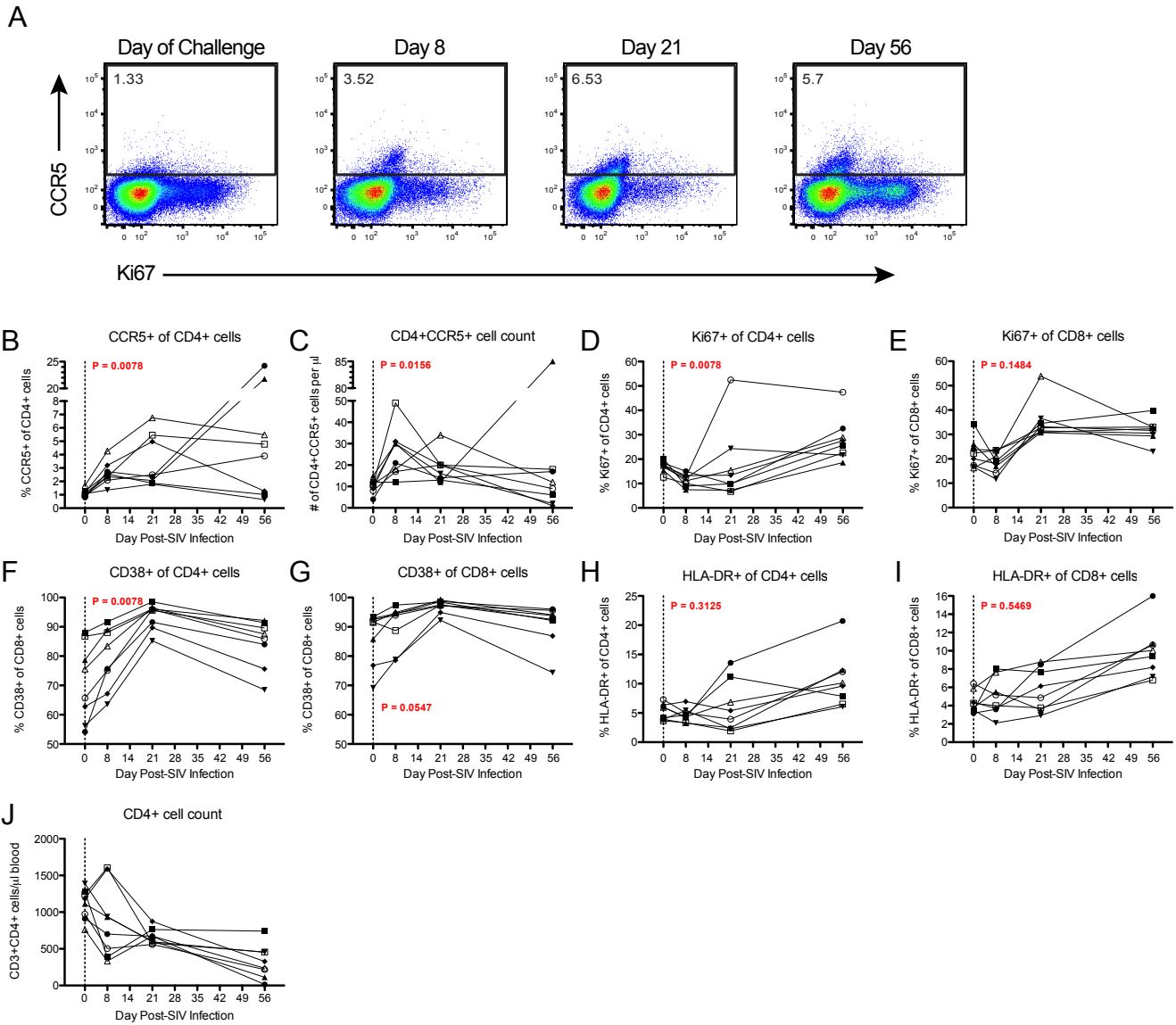

Supplement: S5 Fig — (A) Representative FACS plots gated on peripheral CD4+ cells expressing the HIV/SIV co-receptor CCR5. (B) Frequency of peripheral CD4+ cells expressing CCR5. These frequencies were combined with complete blood counts to determine the (C) absolute number of CD4+CCR5+ cells per μl of blood. (D) Frequency of CD4+ cells expressing Ki67. (E) Frequency of CD8+ cells expressing Ki67. (F) Frequency of CD4+ cells expressing CD38. (G) Frequency of CD8+ cells expressing CD38. (H) Frequency of CD4+ cells expressing HLA-DR. (I) Frequency of CD8+ cells expressing HLA-DR. (J) CD4+ T cell count per microliter of peripheral blood. For all measures of statistical significance, differences between levels from 0 to 8 DPI were evaluated for statistical significance by two-tailed Wilcoxon signed rank testing. (PDF) [file ppat.1006048.s005.pdf]

# SUP FIGURE 6

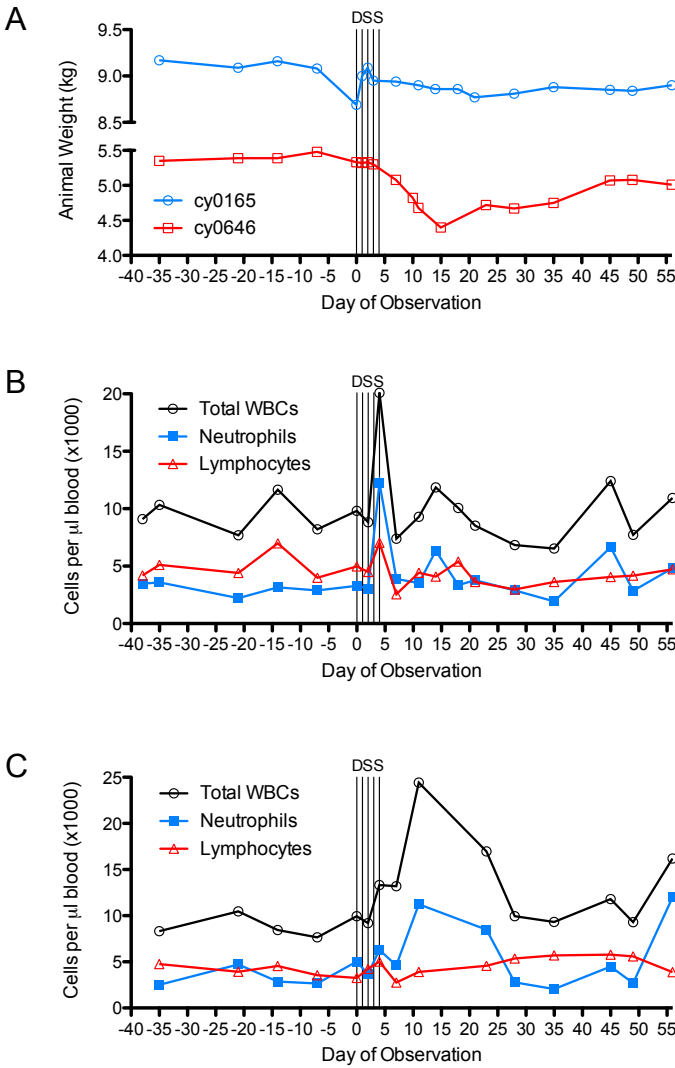

Supplement: S6 Fig — We monitored the weight (A) and hematological health (B and C) of both macaques during dextran sulfate sodium treatment. (PDF) [file ppat.1006048.s006.pdf]
